# Multidimensional analysis of job advertisements for medical record information managers

**DOI:** 10.3389/fpubh.2022.905054

**Published:** 2022-11-04

**Authors:** Pingping Dai, Tongkang Zou, Haiwei Cheng, Zirui Xin, Wei Ouyang, Xiaoqing Peng, Aijing Luo, Wenzhao Xie

**Affiliations:** ^1^Third Xiangya Hospital, Central South University, Changsha, China; ^2^Department of Medical Information, School of Life Science, Central South University, Changsha, China; ^3^Key Laboratory of Medical Information Research (Central South University), College of Hunan Province, Changsha, China; ^4^Clinical Research Center for Cardiovascular Intelligent Healthcare in Hunan Province, Changsha, China; ^5^Second Xiangya Hospital, Central South University, Changsha, China; ^6^Department of Sociology, Central South University, Changsha, China

**Keywords:** job analysis 工作分析, medical record information 病历信息, content analysis 内容分析, visualization map 可视化地图, co-occurrence analyses, competency, competence framework

## Abstract

**Objective:**

The rapid growth of the medical industry has resulted in a tremendous increase in medical record data, which can be utilized for hospital management, aiding in diagnosis and treatment, medical research, and other purposes. For data management and analysis, medical institutions require more qualified medical record information managers. In light of this, we conducted an analysis of the qualifications, abilities, and job emphasis of medical record information managers in order to propose training recommendations.

**Materials and methods:**

From online job posting sites, a sample of 241 job advertisements for medical record information management positions posted by Chinese healthcare institutions were collected. We conducted word frequency and keyword co-occurrence analysis to uncover overall demands at the macro level, and job analysis to investigate job-specific disparities at the micro level. Based on content analysis and job analysis, a competency framework was designed for medical record information managers.

**Results:**

The most frequent keywords were “code,” “job experience,” and “coding certification,” according to the word frequency analysis. The competency framework for managers of medical record information is comprised of seven domains: essential knowledge, medical knowledge, computer expertise, problem-solving skills, leadership, innovation, and attitude and literacy. One of the fundamental skills required of medical record information managers is coordination and communication. Similarly, knowledge and skill requirements emphasize theoretical knowledge, managerial techniques, performance enhancement, and innovation development.

**Conclusion:**

According to organization type and job differences, the most crucial feature of the job duties of medical record information managers is cross-fertilization. The findings can be utilized by various healthcare organizations for strategic talent planning, by the field of education for medical record information managers for qualification and education emphasis adjustment, and by job seekers to enhance their grasp of the profession and self-evaluation.

## Introduction

In 2009, the Chinese government issued Opinions on Deepening the Reform of the Medical and Health System, which called for aggressively advancing medical and health informatics (1). Moreover, the Health China 2030 strategy outline said in 2016 that the standardization of the management and use of electronic health records (EHR) should be fully implemented (2). Among other countries with similar healthcare systems to China, the U.S. federal government enacted Health Information Technology for Economics and Clinical Health (HITECH), which led to the rapid implementation of EHR systems and innovations in health information management services (3). The Australian Commission on Safety and Quality in Health Care (ACSQHC) acknowledged that incomplete medical record files could result in unfavorable incidents involving patients and doctors (4). Medical record data is a fundamental component of reporting in Australian healthcare institutions (5). Clinical practice has demonstrated that medical record data is crucial to the operation of the Diagnosis Related Groups (DRGs) system (6–8). Clinical decision-making is increasingly based on data from medical records (9, 10). Liu Aimin, a Chinese expert on medical record information management, explained that medical record information management refers to the in-depth analysis and physical management of medical records; the extraction of useful information from case data for scientific management; and the provision of high-quality health information services (11). Consequently, positions for medical record information managers have been created in healthcare institutions.

The proficiency of medical record information managers will have an immediate impact on the quality of medical record management. For example, coders must be highly trained in disease classification; otherwise, they may be biased when faced with difficult situations (12). In research conducted by Meghan E. Edmondson, data quality issues accounted for 91% of secondary applications of electronic medical records (13). On the technical side, this is due to the limits of the computer and the EHR system (14), the complexity of coding terminology, and the lack of consensus over coding standards (15). On the organizational side, the main drivers of EHR data quality (16) are the knowledge structure of coders, their degree of education, cultural characteristics, and the engagement and attention of healthcare practitioners and hospital leaders. Human factors, such as missing information (17) and inconsistent information (18), as well as inadequate financial budgets and a lack of incentives (16), are primarily responsible for the behavioral components. Clearly, the quality of the raw data entered by case information managers has a direct impact on the smooth development of subsequent studies and plays a significant role in medical research (14). With the rapid development of big data in medicine, medical record information management focuses more on data mining and utilization, with multiple purposes including epidemiology, precision medicine, and medical screening (19), and competency requirements for medical record information managers have changed (20). Therefore, it is a worthwhile theoretical hypothesis to define the existing competency requirements of medical record information managers.

Chinese medical record information managers now tend to be of poor quality. A recent survey revealed, for instance, that only 61% of medical record information managers in 105 hospitals in China have a bachelor's degree or above, and 15% have not even earned coding certification (21). Up to 60 and 55%, respectively, of medical record quality concerns were due to delayed writing and misfiled or missing first pages (22). In another study (23), flaws in reading medical record, incompetence with coding regulations, and a lack of clinical understanding among medical record information managers were identified as common reasons of coding errors. Medical institutions do not pay sufficient attention to medical record information managers, and insufficient staffing and aging are the primary issues (24). Some medical record information managers are promoted directly from the ranks of registered nurses or medical technicians (25). As an illustration, office personnel, medical statisticians, and nurses may become medical record information managers (26). Li et al. survey indicated that medical coders had poor education, usually college, and a heavy daily burden due to inadequate staffing (27, 28). Tang et al. found a “digital divide” between medical record information managers and medical practitioners due to poor communication and inadequate training (29, 30). A survey of clinical coders in Australia, the US, Canada, and the UK found that lack of formal training, insufficient advanced degrees, and a hectic daily workload were the primary issues (31).

The American Center for Education Statistics defines competency as a combination of skills, abilities, and knowledge needed to perform a specific task (32). Health information management (HIM) is the practice of acquiring, analyzing, and protecting digital and traditional healthcare information critical to providing quality patient care (33). A recent workforce report outlines the conventional tasks of health information management specialists, such as data maintenance, clinical coding, and disease classification. However, it may also play a part in monitoring and management responsibilities (33). Competency frameworks for health informatics are viewed differently in various nations. The Australian Health Informatics Society developed assessment recommendations for health informatics competencies in 2013 (34). The Australian Health Information Education Council categorized the Australian Health Information Management Competency Framework into 45 core competencies in six dimensions (35). The American Medical Informatics Association (AMIA) developed the medical informatics graduate core competencies in 2017, concentrating on three essential areas: health, information science, and social and behavioral science (36). The Canadian Medical Informatics Association also defined 12 core competencies in the field of health information management, representing the necessary knowledge and skills for certification and training of professionals (37). Sapci and Sapci synthesized national standards for health informatics abilities and built a new framework that defines health information competencies into six components: fundamental knowledge; medical information knowledge; medical knowledge application; medical technology application; problem-solving skills; and innovation skills (38). Jinyu described a competency model for medical record information managers based on their professional knowledge and skills, their professionalism, and their personal qualities, with nine secondary and 42 tertiary indicators (39). Although the study is instructive for all medical record information managers, it cannot reflect position-specific variances in terms of specifics. One study evaluating the fundamental competences of coders suggested that coders should be selected with an emphasis on their professional skills, communication skills, and learning and innovation (40). However, it lacked factual data to assess the current state and requirements of the entire medical record information management business thoroughly. With the widespread adoption of electronic health records in the United States, medical scribes have emerged as a prominent new employment whose major function is to alleviate the workload of physicians by handling the entry, output, review, and other auxiliary functions of electronic medical records (41). It may appear that medical scribes are also accountable for the duties of medical report information management. A recent study developed for the first time a core knowledge, skills, and attitudes (KSA) model for medical scribes, which includes three categories of didactic, hands-on learning, and prerequisites (42). Despite the fact that various organizations and scholars have developed core competency criteria for health informatics, they are unable to clarify the discrepancies between job posts on a more detailed level. Therefore, this research will provide a more comprehensive analysis of the job duties, skill gaps, and domain expertise of medical record information managers.

Online job ads are job listings by employers on specialized websites, while online recruiting is the process of identifying and attracting suitable employees over the Internet (43), with basic requirements and job tasks. Job advertising data may give a more complete and effective analysis of the changing labor market circumstances, as well as insightful ideas for the construction of curricula in higher education and training institutions (44). Meyer evaluated the prerequisites and personal competence features of healthcare data scientist and population health management positions by analyzing online job advertising (45, 46). Few researchers have evaluated online job advertisements for medical record information managers. Hence, this work will examine the competencies of medical record information managers based on online job advertisements.

We investigated the current situation and requirements of medical record information managers. By sampling job ads from online sites at a certain time, we thoroughly analyzed the requirements for the competencies and skills of medical record information managers and the subtle differences between positions. We also provided a reference for exploring the development of Chinese medical record information management talents and related policies.

## Method

**Figure 1:**
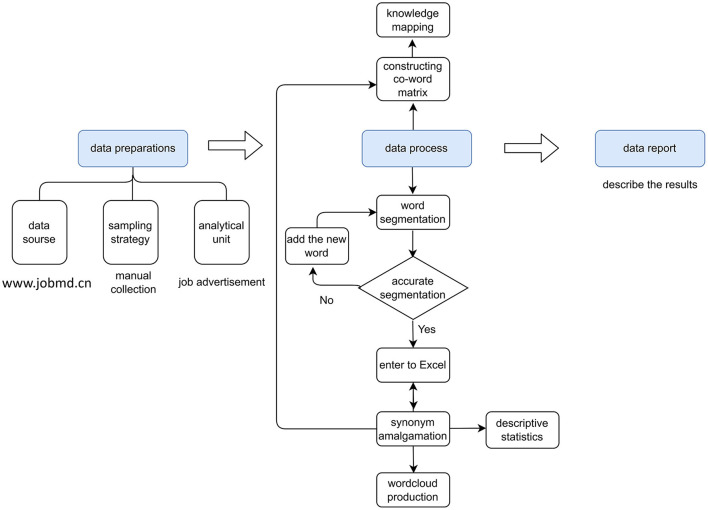
The content analysis process (Edited in 2021/4, China).

In this study, content analysis, word frequency analysis, co-occurrence analysis, and job analysis are used as part of a mixed-methods to qualitatively and quantitatively analyze the content of job ads. Content analysis is the process of examining vast volumes of textual data and describing document content in order to evaluate the frequency and trends of text words and to classify, de-textualize, re-textualize, and assemble text (46–48). Using categorization statistics, content analysis can exhibit a huge quantity of textual content as graphs and charts in order to find potential information in the text, do comparison studies, and generate new ideas. Word frequency analysis is the core of content analysis (49). By examining the text, word frequency can unearth the focus of the text's content; when one or more topic words are repeated in the text, the word can to some extent reflect the text's focus and subject matter; typically, keywords are phrases used to characterize the text's core content (50). Co-occurrence analysis is when keywords exist in multiple articles simultaneously, allowing us to presume that they are related or reflect the same topic. The greater the frequency of their co-occurrence, the stronger the link. Job analysis organizes, extracts, and evaluates a sequence of processes performed on job ads. Figure 1 shows how the content analysis method used in this study was adapted from previous research.

### Data preparation

According to the content analysis procedure, the preparation phase includes three parts: data collection method, sampling strategy, and analysis unit (51). The study data was collected from a variety of medical recruitment websites, including Dingxiang Talent Net (www.jobmd.cn), Medical Talent Net (www.doctorjob.com.cn/), and China Health Talent Net (www.21wecan.com/index.html), among others. Institutions (such as research institutes and public hospitals) and commercial businesses are included in the scope of the recruitment information, which is broadly representative and simple to sample. The sampling period is confined to January to June 2020, and the text of each job ad is preserved in an Excel file for further study. The sampling strategy involved searching the target website for medical record management job ads, including “medical record management,” “medical coding,” or “disease classification” in the job ads. Additional search criteria were not constrained. Job announcements that fit the requirements were inspected and included individually. Duplicate job ads were deleted. Changes in job ads were observed and evaluated during data collection to determine data saturation (i.e., an indication of optimal sample size). Almost no new job ads were confirmed to have reached signal data saturation after 6 months. After processing the raw data, a total of 241 job ads were acquired. The unit of analysis was every job advertisement. Multiple reviews of the data were conducted to gain a better understanding of the entire sample of job ads and the content modules.

### Data processing

The data processing phase consists of data preprocessing, data cleansing, word cloud building, co-matrix construction, and visualization. In the word separation phase, the Python JieBa module is combined with custom word lists to separate the job ad texts. Since the expression of the same term in different texts may vary, it is necessary to combine synonyms for keywords. For instance, “data” and “medical data” are merged into “medical data;” “document writing,” “copywriting,” and “writing skills” are merged into “writing skills;” and “quality control of medical records” and “quality” are merged into “quality control.” After combining synonyms, we ultimately obtained 113 different keywords. We constructed the keyword co-occurrence matrix by the BICOME (V2.01), which included “knowledge,” “skills,” “personal ability,” and “basic quality.” Finally, we visualized and analyzed the keyword co-occurrence matrix by Gephi (V0.9.2).

### Data reporting

In this phase, the results were adjusted and analyzed based on summary statistics and visualization mappings. The variations among the posts were evaluated in a micro view.

## Results

### Content analysis summarized statistics

Healthcare institutions in China are ranked from lowest to highest on a three-tier scale. As tertiary hospitals are able to drive regional health development plans and are among the most advanced in China in terms of professionalism, secondary and higher hospitals lead the health region in terms of technical level (52). This study collected job ad data from 241 healthcare-related institutions in total. Recruitment for medical record information management was predominantly found in hospitals, with public hospitals accounting for ~49.8%, private hospitals for 43.6%, and biological and pharmaceutical companies accounting for 6.8%. Regarding hospital classification, tertiary and higher hospitals were the primary employers, accounting for approximately half of the job ads. Consequently, the study's findings are representative (Table 1).

**Table 1:** Sample descriptive statistics (*N* = 241).

	**Frequency**	**Percentage (%)**
**Nature of the recruiter**		
Public hospitals	120	49.8
Private hospitals	105	43.6
Biological and pharmaceutical enterprises	2	0.8
Other	14	5.8
Total	241	100.0
**Hospital rating**		
Tertiary hospital and above	115	47.7
Secondary hospital	67	27.9
First-Level hospital	6	2.5
Other	53	21.9
Total	241	100.0
**Educational background**		
Technical secondary school and above	2	0.83
College and above	108	44.81
Bachelor and above	105	43.57
Master and above	16	6.64
Doctor	1	0.41
Unlimited	9	3.73
Total	241	100.00
**Job category**		
Coder	75	31.12
Medical services manager	1	0.41
Medical record manager	114	47.30
Medical record statistician	16	6.64
Medical record quality controller	8	3.32
Medical affairs section officer	2	0.83
Data standardization project engineer	1	0.41
Medical record office technician	2	0.83
Healthcare DRGs information manager	1	0.41
Not listed	21	8.71
Total	241	100.00
**Work experience requirements**		
Freshmen	9	3.73
1–3 years (including 3 years)	64	26.56
3–5 years (including 5 years)	18	7.47
5–10 years (including 10 years)	9	3.73
More than 10 years	2	0.83
Unlimited	139	57.68
Total	241	100.00
**Title requirements**		
Junior and above	40	16.6
Intermediate and above	6	2.5
Associate senior and above	3	1.2
Unlimited	192	79.7
Total	241	100.0
**Professional requirements**		
Health management	131	34.7
Clinical medicine	102	27.0
Public health and preventive medicine	64	16.9
Management science and engineering	18	4.8
Public management	17	4.5
Other	46	12.2
Total	378	100.0

**Figure 2:**
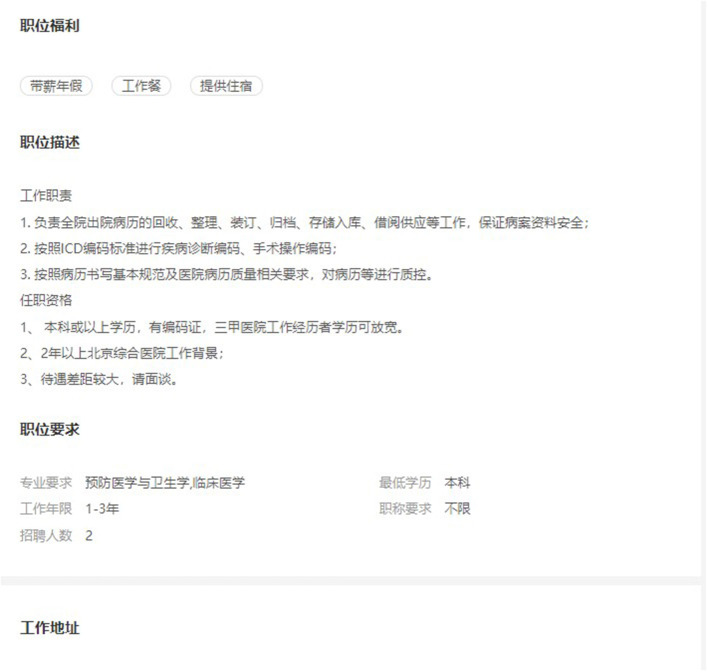
Example of original job advertisement (Edited in 2022/6, China).

Figure 2 is an example of the original job advertisement, including the job description, requirements, work location, and benefits. The job description, requirements, title, and work location parts were extracted and analyzed the most. The majority of the job postings are for coders and medical record managers (above 70%), with the remainder for medical record statisticians and quality controllers. There are very few recruiters who require a PhD degree, and the majority of hospitals' educational requirements for medical record information managers are specialty and bachelor's degrees. Some jobs require only a secondary school education or no formal education at all. Health management and clinical medicine were the most common majors, followed by public health and preventive medicine. More than half of the recruiters did not require years of experience; of the other half, the majority required 1–3 years of experience (26.56%), and very few required more than 10. Most employers expected their employees to be junior level and above, with only nine job ads (*N* = 241) for medium and senior titles. Most employers did not expect their employees to possess a job title.

### Analysis of keyword frequency

Table 2 displays the keywords that occur more than twice. After excluding the subject word “medical records,” “coding” appeared 106 times, followed by “job experience” (87 occurrences), “coding certification” (85 occurrences), “ICD” (81 occurrences), and “medical records management” (56 times).

**Table 2:** Keywords after splitting (Frequency > 2).

**Keyword (Chinese/English)**	**Frequency**	**Percentage (%)**
编码 (Coding)	106	10.91
工作经验 (Work experience)	87	8.95
编码资格证(Code certificate)	85	8.74
ICD (International classification of diseases)	81	7.33
病案管理 (Medical record management)	56	5.76
沟通能力 (Communication skills)	37	3.81
职称证书 (Title certificate)	35	3.60
医学统计 (Medical statistics)	30	3.09
质控 (Quality control)	22	2.26
办公软件 (Office software)	22	2.26
责任心 (Responsibility)	20	2.06
医疗 (Medical)	17	1.75
团队意识 (Team awareness)	17	1.75
医务科 (Medical department)	16	1.65
法律法规 (Laws & regulations)	14	1.44
计算机 (Computer)	14	1.44
DRG(DRG)	12	1.23
医学知识 (Medical knowledge)	10	1.03
服务意识 (Service awareness)	10	1.03
统计学 (Statistics)	9	0.93
年龄 (Age)	9	0.93
病案信息技术证(Medical record information technology certificate)	9	0.93
亲和力 (Affinity)	8	0.82
科研 (Scientific research)	8	0.82
临床经验 (Clinical experience)	8	0.82
保密意识 (Confidentiality)	8	0.82
规章制度 (Rules & regulations)	7	0.72
敬业心 (Dedication)	7	0.72
教学 (Teaching)	7	0.72
组织管理能力 (Organizational management capabilities)	7	0.72
写作能力 (Writing ability)	7	0.72
完整性 (Completeness)	7	0.72
职业道德 (Professional ethics)	6	0.62
医疗数据 (Medical data)	5	0.51
学习创新能力 (Learning & innovation)	5	0.51
数据库 (Database)	5	0.51
统计资格证(Statistical qualification certificate)	4	0.41
知识讲座 (Knowledge lectures)	4	0.41
执业医师证(Practicing physician certificate)	4	0.41
临床业务 (Clinical business)	4	0.41
反馈 (Feedback)	4	0.41
医院管理 (Hospital management)	4	0.41
数据分析能力 (Data analysis capabilities)	4	0.41
医保(Health care)	3	0.31
医技 (Medical technology)	3	0.31
隐私(Privacy)	3	0.31
护理 (Nursing)	3	0.31
应届生 (Graduates)	3	0.31
病案统计科 (Medical record statistics department)	3	0.31

**Figure 3:**
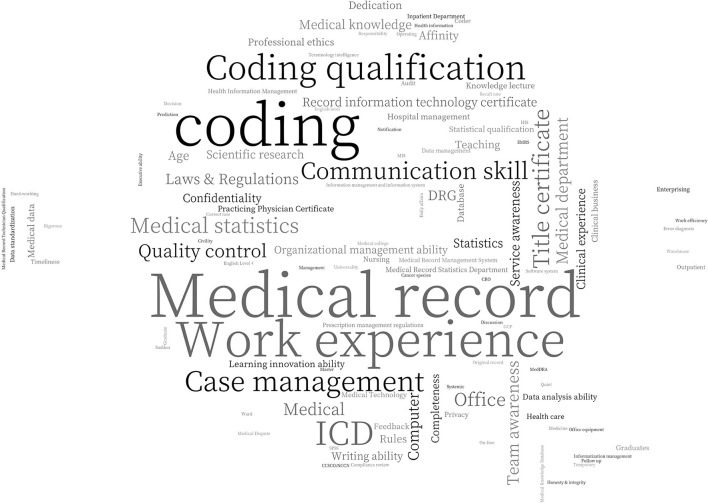
Word cloud mapping of keyword frequency analysis (Edited in 2021/4, China).

Figure 3 is a word cloud mapping constructed on the frequency of keywords following word separation. Different node sizes correspond to varying weights; the frequency of a keyword increases as its node size. Certificate requirements for medical information managers consist of the terms “coding qualification,” “medical practitioner certification,” and “medical record information technology qualification.” Figure 3 is a word cloud mapping constructed on the frequency of keywords following word separation. Different node sizes correspond to varying weights; the frequency of a keyword increases as its node size. Certificate requirements for medical information managers consist of the terms “coding qualification,” “medical practitioner certification,” and “medical record information technology qualification.” “Disease classification” implies that the most important skills for medical record information managers are coding and disease classification. The terms “Medical statistics,” “statistical qualification,” “statistics,” “SPSS,” and “database” suggest a requirement for data processing abilities among medical record information managers. “English competence” shows the foreign language skills required by a few job ads for medical record information managers, and “laws and regulations” suggests that medical record managers will be involved in medical and legal matters. Furthermore, they must possess the essential attributes of “communication skills,” “team spirit,” and “responsibility.”

### Keyword co-occurrence analysis

**Figure 4:**
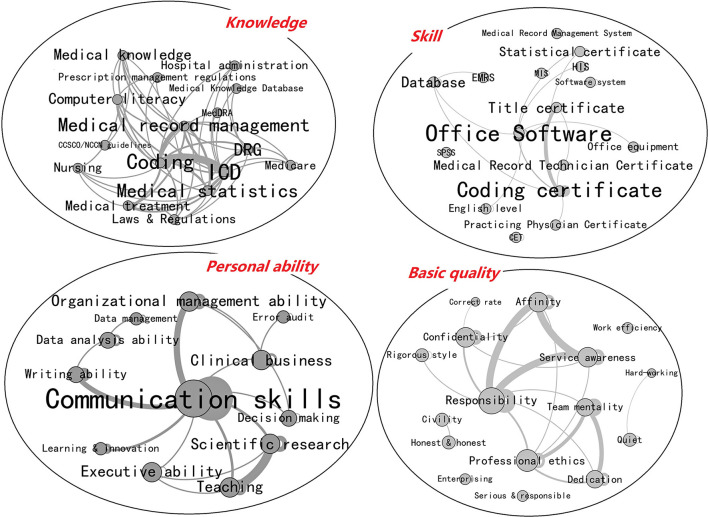
Visualization map of co-occurrence analysis (Edited in 2021/4, China).

We created respective co-occurrence matrices for “knowledge,” “skills,” “personal ability,” and “basic job quality.” As depicted in Figure 4, we utilized Gephi to make a visualization map with proportional node size, color depth, and keyword weights.

In the “Knowledge” part, the three nodes “Coding,” “ICD,” and “Medical Record Management” have the closest correlation. It is obvious that understanding of coding and medical record management are the most essential qualifications required by employers of their employees. In the figure, “Statistics” and “ICD” are interconnected, demonstrating that coding and statistics are complementary. Legal knowledge is necessary for medical disputes and daily tasks such as medical record coding and management.

The “Skills” part consists primarily of office software, certifications, and databases. A medical coding certificate is the most fundamental certification for medical record information managers. Many organizations additionally require computer certification II because they require employees to be fluent in Excel, PowerPoint, and Word. SPSS proficiency and a statistical qualification match to the requirements of medical record statistics. Some employers require staff to possess a Medical Practitioner Certificate, demonstrating that they value employees' medical background and knowledge.

In the “personal ability” part, “communication skills” are the main focus, which most closely related to “organizational management skills,” “clinical business,” and “writing skills,” representing employers' core skill requirements. In managerial jobs, competencies in organizational management, clinical business, and decision-making are more essential than in other positions. When it comes to filing and reporting medical records, writing and data analytic abilities are indispensable.

In the “Basic Quality” section, “Responsibility” is the core of two basic quality requirements: “Confidentiality Awareness,” “Affinity,” and “Service Consciousness,” as well as “Team Consciousness,” “Professional Ethics” and “Professionalism.” Employers have varying expectations for fundamental employee qualities based on the various job types. For management roles, candidates must possess “affinity” and “responsibility,” but for technical posts, “professional ethic,” “dedication,” and “professionalism” are required. Concurrently, medical records include patient privacy, so a strong confidentiality is basic for all medical record information managers.

### Job analysis of typical positions

Table 3 displays the job duties, knowledge, and skill requirements for four positions based on job ads, excluding job categories without postings.

**Table 3:** Job analysis of typical positions.

**Job title**	**Duties**	**Tasks**	**Prerequisite knowledge, skills, experiences**	
			**Knowledge (frequency)**	**Skills (frequency)**
Medical record coder (*N* = 75)	Medical record management	Storing, copying, and maintaining medical records Inputting and managing medical record homepage Implementing laws and regulations on medical record management Observing the confidentiality of medical records	Coding knowledge (39), Medical record management knowledge (6), Medical knowledge (6), Office software knowledge (5), Computer knowledge (4), Statistics knowledge (1), Knowledge of DRGs (2),	Communication skills (2), Learning innovation ability (2), Data analysis ability (1), Resilience (1), writing ability (1)
	Medical record coding	Responsible for coding medical record	Pharmaceutical knowledge (1),	
		Responsible for writing patient information card	English knowledge (1), Legal knowledge (1)	
		Responsible for coding and inputting discharge medical record		
		Registering coding problems and feedback		
	Medical record quality control	Responsible for the final quality control of medical records		
		Reviewing the quality of medical records		
		Checking and altering duplicate name and card		
		Responsible for the quality control of the medical record homepage		
		Checking the integrity of medical records		
	Medical record statistics	Responsible for medical record statistics		
		Responsible for homepage analysis of medical records		
	Scientific research & teaching	Assisting in scientific research		
		Assisting in training, meeting records		
Medical record manager(*N* = 114)	Medical record management	Responsible for medical records recovery, review, sorting, indexing, etc Responsible for medical records inquiries, copying, and borrowing Providing medical records for others Cooperating with medical record spot check Responsible for the information management of medical records	Coding knowledge (47), Medical record writing knowledge (24), Medical record management knowledge (24), Statistical knowledge(23), Office software knowledge (12), Legal knowledge (7), Hospital management knowledge	Communication skills (25), Learning innovation ability (4), Organizational management ability (4), Publicity and expression ability (3), Team awareness (1), Resilience (1), Service awareness (1), Writing ability (1)
	Hospital management	Responsible for department business management	(5), Computer knowledge (5),	
		Supervising departments to abide by the medical records management rules	Data mining knowledge (1),Data management knowledge (1),	
		Managing medical records room	Medical knowledge (1),	
		Regular communication training with clinical	Medical care knowledge (1), DRGs (1)	
		Establishing follow-up procedures		
		Assisting health care management		
		Responsible for hospital infection and quality management		
		Assisting clinical business and quality management		
		Assisting in handling medical disputes and preventing accidents		
		Implementing hospital rules and regulations		
	Document management	Completing medical documents and report management		
		Editing various compilation materials		
	Medical record quality control	Responsible for the quality control of the medical record homepage		
		Reviewing the quality of medical records		
		Responsible for the final quality control of medical records		
		Checking the writing of medical records and proposing suggestions		
	Data statistics	Responsible for medical information statistics and reporting		
		Related data report statistics		
		Conducting statistical analysis of large sample data		
	Medical record coding	Responsible for coding medical record		
Medical record statistician (*N* = 16)	Medical record management Data collection and statistics	Responsible for the retrieval, sorting, filing, etc. of medical records Collecting raw data from departments Collecting, sorting, and checking of statistical data	Coding knowledge (5), Medical record management knowledge (4), Statistical knowledge (4), Computer knowledge (2),	Communication skills (2), Organizational management ability (1), Team awareness (1)
		Statistical consultation and information feedback	Office software knowledge (2), SQL (1),	
		Providing statistical results	Hospital management	
		Compiling with statistical rule and method	knowledge (1), Knowledge of DRGs (1)	
		Supervising the medical statistics of departments		
		Compiling and analyzing statistical reports		
		Statistics of medical efficiency and medical quality		
	Discipline development	Discipline construction, operation and development		
		Disciplinary team formation and talent echelon construction		
		Formulating and implementing disciplinary development plans		
		Promoting subject professional ability and service level		
Medical record quality controller (*N* = 8)	Medical record quality control	Quality control of medical records Scoring inspection medical record quality control Summarizing and reporting the inspection results Discovering and solving medical record writing problems Modifying the wrong medical record	Knowledge of medical record writing (3), Coding knowledge (2), Legal knowledge (1), Medical knowledge (1), Management knowledge (1), Statistical knowledge (1), Knowledge of DRGs (1),	Communication skills (4), Publicity and expression ability (1), Resilience (1), Decision-Making ability (1), Team awareness (1), Organizational management ability (1)
	Personnel training	Training new physicians on medical record writing knowledge	Medical care knowledge (1)	
		Convene a medical quality control staff meeting		
		Helping physicians develop good medical record writing habits		
		Conducting medical record writing knowledge lectures		
		Training, guiding, and supervising of clinical medical record specifications		

### Medical coder

Medical coders are responsible for coding, managing, and quality-controlling medical records and, in some situations, assisting with medical record statistics and scientific research instruction. The predominant educational qualification for this position is a bachelor's degree or above, with a few public tertiary hospitals requiring a master's degree and a few freshmen available. Coding knowledge is typically required by employers, followed by understanding of medical record management and medicine. Coders should strive to develop their communication and coordination skills, as well as their ability to learn and innovate.

### Medical record manager

Medical record managers' critical areas of responsibility include medical record management and hospital management, with hospital management relating primarily to communication and business transactions with clinical departments (Table 3). Managers of medical records must master all the procedures of medical record management, and their knowledge areas are more comprehensive than others. Their educational requirements are low. For instance, more than half of companies require a specialty or higher. Medical record managers must have excellent communication and coordination skills, as well as a strong sense of responsibility, affinity, and service consciousness. In general, the experience requirements for medical record managers are greater, ranging from 1 to 3 years, with a few positions requiring 5–10 years of experience.

### Medical record statistician

As seen in Table 3, medical record statisticians are primarily responsible for the department's data statistics. Few public tertiary hospitals expect them to be accountable for developing departmental disciplines, which necessitates a higher level of competency, extensive work experience (more than 10 years), and an intermediate title. Higher standards for computer proficiency, including expertise in Excel and database skills, apply to medical record statisticians. Their main places of work are public tertiary hospitals, and about one-third of them now require a master's degree.

### Medical record quality controller

Medical record quality controllers are primarily responsible for medical record quality control and coder training. They are knowledgeable in writing medical records. In addition, medical record quality controllers possess a deeper understanding of the law, healthcare, and DRGs and are required to remain current on the most recent national quality control standards. Experience is more important than education for them, and most employers want at least 5 years of professional experience, along with good communication and expression skills.

Table 4 illustrates the distinctions between the four typical positions in public hospitals, private hospitals, and biopharmaceutical companies. Overall, it appears that biopharmaceutical businesses employ the fewest medical record managers and coders. Coders have expanded responsibilities inside the organization, including medical data analysis and the development of a medical knowledge base. Medical case managers are increasingly prevalent in private institutions, where employers expect greater expertise and abilities. Quality controllers and medical record statisticians are less frequently hired positions with comparable requirements for all employers.

**Table 4:** Job analysis of typical positions (classification by different organizations).

**Organization type**	**Job title**	**Duties**	**Prerequisite knowledge, skills, experiences**	
			**Knowledge**	**Skills**
Biological and pharmaceutical corporation (*N* = 9)	Medical record coder (*N* = 6)	Coding; Medical Record quality control; Medical record management, Data analysis, Terminology intellectualization, Medical knowledge base, Medical professional communication, Clinical assistance	Coding knowledge (4), Medical knowledge (5), Pharmacy knowledge (1), DRGs knowledge (1), Healthcare knowledge (1)	NA
	Medical record manager (*N* = 3)	Medical record management, Coding, Staff development, Data analysis, Medical record quality control, Database operation	Office (1), Legal knowledge (1), Coding knowledge (1), Statistical knowledge (1), Database knowledge (1), Medical record management knowledge (1), Medical knowledge (1), Medical record writing knowledge (1), Computer knowledge (1)	Communication and Coordination ability, Learning and Innovation ability, Service consciousness, Cooperation consciousness
Public hospital (*N* = 110)	Medical record coder (*N* = 50)	Coding, Medical record management, Medical record quality control, medical record statistics, Document processing, Scientific research and teaching	Coding knowledge (27), Medical record management knowledge (5), Statistics knowledge (1), DRGs knowledge (1), Medical knowledge (3), Medical record writing knowledge (3), Office (1)	Learning and Innovation ability, Enterprising, Adaptability, Communication and Coordination ability, Cooperation consciousness, Dedication, Writing ability
	Medical record manager (*N* = 46)	Coding, Medical record management, Medical record quality control, Medical record statistics	Coding knowledge (17), Medical record management knowledge (4), Statistics knowledge (4), Data mining knowledge (1), Medical record writing knowledge (7), Computer knowledge, Healthcare knowledge (1), Legal knowledge (1), Office (1)	Organization and Management ability, Communication and Coordination ability, Learning and innovation ability, Cooperation consciousness, Service consciousness, Dedication
	Medical record statistician (*N* = 12)	Coding, Medical record management, Data collection and statistics, Discipline development, Medical record quality control, Departmental management	Medical record management knowledge (2), Medical record writing knowledge (1), Coding knowledge (3), DRGs knowledge (1), Statistics knowledge (2), Computer knowledge (1), Hospital management knowledge (1)	Organizational and management ability, Teamwork awareness
	Medical record quality controller (*N* = 2)	Medical record quality control, Staff training, Medical record management	Legal knowledge (1), Medical knowledge (1), Management knowledge (1), Coding knowledge (1), Medical record management knowledge (1)	Communication and coordination ability, Adaptability, Decision-Making ability, Organization and Management ability, Teamwork awareness
Private hospital (*N* = 97)	Medical record coder (*N* = 20)	Medical record management, Coding, Medical record statistics, Trainings, Project management, Scientific research and teaching	Coding knowledge (9), Computer knowledge (4), Office (4), DRGs knowledge (1), Medical record writing knowledge (3), Legal knowledge (1), Medical record	Learning and innovation ability, Dedication, Cooperation consciousness, Interpersonal ability, Organization and management ability,
			management knowledge (1), English knowledge (1)	Communication and coordination ability, Data analysis ability, Responsibility
	Medical record manager (*N* = 67)	Medical record management, Coding, Hospital management, Document management, Medical record statistics, Decision-Making	Coding knowledge (30), Medical record management knowledge (31), Medical record writing knowledge (17), Legal knowledge (5), Statistics knowledge (18), Computer knowledge (6), Hospital management knowledge (5), Office (9), Healthcare knowledge (1), DRGs knowledge (1), Database knowledge (1)	Communication and Coordination ability, Learning and Innovation ability, Service awareness, Responsibility, Affinity, Organization and Management ability, Writing Ability, Decision-making ability, Cooperation, Service Awareness, Adaptability
	Medical record statistician (*N* = 4)	Data collection and statistics, Medical record management, Medical statistics	SQL knowledge (1), Statistics knowledge (2), Coding knowledge (1), Medical knowledge (1), Computer knowledge (1), Medical record management knowledge (1), Office (2)	Communication and coordination ability, service awareness, Responsibility, Affinity
	Medical record quality controller (*N* = 6)	Medical record quality control, Staff training	Medical record writing knowledge (4), Coding knowledge (2), Statistics knowledge (1), DRGs knowledge (1), Healthcare knowledge (1)	Communication and Coordination ability, Teamwork awareness, Cooperation awareness, Publicity and Expression ability

As shown in Table 5, we outline the key competences of different levels of medical record information managers across seven dimensions: basic knowledge; medical knowledge; computer knowledge; problem-solving ability; leadership; innovation ability; and attitude and literacy. There are a total of 29 subdomains. The competencies overlap with the distinctions between positions, even though the emphasis varies considerably by position. No matter what the role is, it is evident that communication and coordination skills, information system understanding, and responsibility are crucial.

**Table 5:** Core competencies of medical record information managers.

**Core competencies**		**Medical record**	**Medical record**	**Medical record**	**Medical record**
		**coder**	**manager**	**statistician**	**quality controller**
**Category**	**Sub-category**				
Essential knowledge	Legal knowledge	++	+++	+	++++
	English knowledge	++	+	+	+
	Statistics knowledge	+++	++++	+++++	+
Medical knowledge	Medical knowledge	++++	++	++	+++
	Healthcare knowledge	+	+++	+	++++
	Pharmacy knowledge	++++	++	+	++++
	Hospital management knowledge	+	++++	+++	+++
	Coding knowledge	+++++	++++	+++	+++
	Medical record management knowledge	+++	+++++	+++	+
	Medical record writing knowledge	+	++++	+	+++++
Computer expertise	Office	+++	+++	+++++	++
	Database knowledge	+	+	++++	+
	Data mining knowledge	++	+++	++++	+
	Information system knowledge	++++	++++	++++	++++
Problem-solving skills	Data analysis ability	+++	++	++++++	+++
	Writing ability	++++	+++	++	++
Leadership	Communication and coordination ability	+++++	+++++	++++	+++++
	Organization and management ability	+	++++	+++	++++
	Publicity and expression ability	++	+++	+++	++++
	Decision-Making ability	+	+++	+	+++
	Teamwork ability	+++	++++	++++	++++
Innovation	Learning and Innovation ability	++++	+++	++	+
	Adaptability	+++	+++	++	+++
Attitude and literacy	Responsibility	++++	+++++	++++	++++
	Enterprising	+++	++	+++	++
	Dedication	+++++	++++	++++	+++
	Service awareness	++	+++++	++++	++
	Affinity	++	+++++	+++++	+++
	Confidentiality	++++	++++	++	++

The dark blue is the most core competencies for medical record information managers. And the light blue is the second.

## Discussion

Based on online job advertisements, our study assessed the qualifications, knowledge, abilities, and major areas of responsibility of medical record information managers and created an outline of their core competences. These positions are frequently related to strategic initiatives such as performance enhancement and discipline development. The management of medical record information involves multiple occupations, and a single position assessment cannot adequately reflect the competencies required by the entire profession (40). In order to thoroughly reflect the competency needs of various roles and the variances between them, we analyzed empirical data from job advertisements based on individual occupations. As healthcare reform advances, medical record information managers play a greater role in enhancing healthcare quality management and preventing healthcare resource waste. The transition from paper to electronic records has also emphasized the need for enhanced training for medical record information managers (53). Consequently, this research is crucial for strategic planning, talent evaluation, and curriculum creation.

Similar to Meyer's (46) job analysis of healthcare data scientists, our study reveals that a variety of healthcare organizations are the primary recruiters of medical record information managers. In tertiary hospitals with higher levels of work, where candidates must have more experience and higher titles, highly qualified hires are typically located. Employers still appear to view coding, medical record administration, and statistics as essential qualifications for medical record information management jobs, notwithstanding the rarity of senior-level recruitment. However, Lucyk et al. (30) and Resslar et al. (54) revealed that the qualifications of the coder and communication between physicians and coders had a greater impact on the quality of coding. Therefore, organizations must prioritize the recruitment of diverse talents. Coders are hired more frequently by public hospitals, while medical record managers are recruited more frequently by private hospitals. Competencies differentiate them more than their very identical knowledge and skill requirements. Several studies have shown that tertiary institutions can hire medically trained individuals as medical record managers (55). It could be because public hospitals place a greater emphasis on employee knowledge and personal qualifications. In contrast, private hospitals value the innate qualities of their personnel and compensate for their lack of knowledge through training. Commonly, employers require medical record information managers to hold relevant certificates. Nevertheless, prior research has demonstrated that a small number of them are unqualified (21).

We established a new competency framework that integrates fundamental to advanced health information management talents and evaluates the current job market requirements for medical record information managers' competencies in seven dimensions: basic knowledge, medical knowledge, computer expertise, problem-solving skills, leadership, innovation, and attitude and literacy. The knowledge structure of medical record information managers is comprised of basic knowledge, medical knowledge, and computer literacy. In addition, problem-solving abilities outside of medical record management and coding do not develop much during the period of study. Practice is required to strengthen their ability to apply their knowledge. It is also consistent with the findings of other research that emphasizes the significance of practice (56), where both theoretical knowledge and practical expertise in medical record information management can be acquired (57). Leadership focuses on the function within the team, whereas innovation skills show the initiative of the individual at work. Similar to the findings of earlier studies, innovative skills cannot be included in the curriculum (38).

Our competency framework for medical record information managers rigorously represents the competencies required for different positions, increasing to a certain extent the competency framework for health information management personnel in many global organizations. Previous research (40) has highlighted professional skills, communication and teamwork, and the ability to learn and innovate as the most essential coding competencies. We assume that coders' attitudes and literacy are of equal importance and that personal attributes such as responsibility, devotion, and confidentiality can reflect employers' needs. Some studies (39) have also categorized the total capabilities of medical record information managers into three components: professional knowledge and skills; occupational attributes; and personality factors. Nevertheless, our research reveals subtle distinctions between positions. Similar to the findings of Corby et al. (42), our findings indicate that medical knowledge is the foundation of knowledge for medical record information managers. We described the ability to communicate, collaborate, organize, and manage as leadership, which is a vital quality for medical record information managers to possess outside the scope of their employment. Leadership in clinical care also appears regularly in competency frameworks, where communication and collaboration can aid in patient understanding (58), and where leadership plays a crucial role in promoting clinical effectiveness (56).

Cross-pollination of duties is an essential characteristic of medical record information management. Previous research has also demonstrated that the variety of abilities possessed by medical record information managers is closely linked to their intrinsic motivation, which means that job enrichment increases their excitement and interest in their work (59). The combination of jobs and abilities varies with employment level. Employers place a premium on communication and coordination abilities, which are fundamental competencies for medical record information administrators. It refers to the duties and responsibilities of medical record information management, which require frequent interaction with clinical departments. Numerous studies demonstrate that efficient communication with physicians is the key to avoiding a variety of difficulties (12, 29, 60). Medical record managers need to be good at making decisions and know a lot about the law. More experienced medical record managers should be able to help the department make important decisions.

Our findings show that medical record information managers should acquire extraordinary professional skills, particularly in medical record coding and management, hospital administration, performance enhancement, teaching and training, and discipline development. It suggests that knowledge and abilities in these areas should be provided in academic programs so that medical record information managers better prepare for the workforce.

The curriculum requirements for medical record information management education and training programs can be determined by referencing our research findings. Our findings suggest that a curriculum centered on theoretical foundations (knowledge of coding, medicine, and statistics), management methods and principles (medical record management, hospital management methods), and innovative development (disciplinary development and talent cultivation) can meet the critical knowledge and skills needed by medical record information managers in order to perform their jobs, with additional knowledge application skills developed through extensive practice. These findings can be used to make educational programs and help medical record managers choose courses and directions for their career growth.

## Limitation

In this study, we collected data from online labor websites, the original data source, within a limited period, which may have caused certain limitations. (1) The sample size was small, and the collection of data was restricted to a short time span, which may not adequately reflect the latest job requirements. (2) No interviews with medical record information managers were conducted; future research may combine interviews and questionnaires for further assessment.

## Conclusion

This study identifies the crucial importance of medical record information managers and organizational recruiting priorities. The findings emphasize the necessity for periodic assessment of job advertisements, promote awareness of neglected knowledge and abilities in healthcare organizations, enable medical record information administrators to assess their competencies thoroughly, and assist in the design of educational programs.

## Data availability statement

Please contact the corresponding authors for the original research data.

## Author contributions

PD, AL, and WX conceived and designed this study. PD and TZ made great contributions to data collection and processing. TZ was the main contributor to data mapping and processing and wrote and revised the article under the guidance of PD, HC, ZX, and WO. PD, TZ, HC, ZX, WO, and XP regular discussions to modify the article. All authors were involved in the writing or improvement of the article and final approval for the upcoming edition.

## Funding

This study was supported by the Science and Technology Plan Project of Changsha (Grant No. kq1901133) and Natural Science Foundation of Changsha City: Research on tertiary hospitals Internet + Medical health service quality evaluation and user adoption (Grant No. kq2014270).

## Conflict of interest

The authors declare that the research was conducted in the absence of any commercial or financial relationships that could be construed as a potential conflict of interest.

## Publisher's note

All claims expressed in this article are solely those of the authors and do not necessarily represent those of their affiliated organizations, or those of the publisher, the editors and the reviewers. Any product that may be evaluated in this article, or claim that may be made by its manufacturer, is not guaranteed or endorsed by the publisher.
